# Implementation and Experiences of Telehealth: Balancing Policies With Practice in Countries of South Asia, Kuwait, and the European Union

**DOI:** 10.2196/30755

**Published:** 2022-02-08

**Authors:** Oommen John, Suptendra Nath Sarbadhikari, Thanga Prabhu, Ashvini Goel, Alexander Thomas, Sunil Shroff, Fazilah Allaudin, Chaminda Weerabaddana, Dari Alhuwail, Udaya Koirala, Jayalal Johnrose, Patricia Codyre, Andy Bleaden, Shubnum Singh, Shuchin Bajaj

**Affiliations:** 1 George Institute for Global Health University of New South Wales New Delhi India; 2 Prasanna School of Public Health Manipal India; 3 The George Institute for Global Health India New Delhi India; 4 St Johns Health Innovation Foundation Bengaluru India; 5 Telemedicine Society of India Lucknow India; 6 Association of Healthcare Providers India New Delhi India; 7 Madras Medical Mission Chennai India; 8 Planning Division, Ministry of Health Kuala Lumpur Malaysia; 9 Ministry of Health Colombo Sri Lanka; 10 Information Science Department Kuwait University Kuwait City Kuwait; 11 Health Informatics Unit Dasman Diabetes Institute Kuwait City Kuwait; 12 Telemedicine Society of Nepal Kathmandu Nepal; 13 Indian Medical Association New Delhi India; 14 Digital Health and Innovation World Health Organization Regional Office for South-East Asia New Delhi India; 15 European Connected Health Alliance Padfield, High Peak United Kingdom; 16 National Healthcare Council Confederation of Indian Industry New Delhi India; 17 Ujala Cygnus Healthcare Services New Delhi India

**Keywords:** telehealth policy and practice, implementation lessons, challenges in scaling up, capacity building of human resources, data privacy, telehealth, health policy, telemedicine, implementation, challenges, human resources, digital health, data security

## Abstract

This viewpoint summarizes the discussion that occurred during the “Translating Policy to Practice in Telehealth–Lessons from Global Implementation Experiences” panel that was held virtually at Telemedicon2020, December 18-20, 2020. This panel brought together policy and implementation experts from some countries of South Asia, Kuwait, and the European Union to share their experiences in the development and implementation of telehealth standards and of the scale up of telehealth interventions within health systems. Several common themes arose from the discussion, including the significant role of people; encouragement by respective government policymakers; addressing concerns, particularly related to privacy, confidentiality, and security; and capacity building of human resources. These are discussed in turn, along with the future directions identified by the panelists, which emphasized the need for active encouragement toward the adoption and diffusion of digital health in general and of telehealth in particular. All stakeholders, ranging from governmental policymakers to common citizens, need to come together to build trusting partnerships to realize the advantages offered by telehealth.

## Introduction

This paper summarize the discussion that occurred during the “Translating Policy to Practice in Telehealth–Lessons from Global Implementation Experiences” panel held virtually at Telemedicon2020 [1], the annual conference of the Telemedicine Society of India (TSI), from December 18 to December 20, 2020. This panel brought together policy and implementation experts from across the globe to share their experiences in the development and implementation of telehealth standards and of scaling up telehealth interventions within health systems. The panel composition is given in the Table 1. Each panelist was asked to (1) trace the evolution of telemedicine in their respective countries, particularly with reference to the COVID 19 pandemic; (2) describe the current policies guiding telemedicine; (3) describe the actual use and adoption of telemedicine within their countries; and (4) identify future directions in the adoption and diffusion of telehealth.

The COVID-19 pandemic has accelerated the role of various digital health interventions, including telehealth, in supporting health services delivery [2]. Telehealth includes a broader scope of remote health care services than telemedicine. Telemedicine refers specifically to remote clinical services, whereas telehealth can refer to remote nonclinical services (administrative/educational). The Global Strategy for Digital Health (GSDH) 2020-2025 [3] retains this definition of telemedicine as:

The delivery of health care services, where distance is a critical factor, by all health-care professionals using information and communications technologies for the exchange of valid information for diagnosis, treatment and prevention of disease and injuries, research and evaluation, and the continuing education of health care workers, with the aim of advancing the health of individuals and communities.

The GSDH 2020-2025 generally defines digital health as “the field of knowledge and practice associated with the development and use of digital technologies to improve health” [3]. This definition is extended on page 13 of the document as:

This definition encompasses eHealth, in line with that in document EB142/20 on mHealth, noted by the Executive Board at its 142nd session (see document EB142/2017/REC/2, summary records of thirteenth meeting, section 2), which stated that “Today the term digital health is often used as a broad umbrella term encompassing eHealth as well as developing areas such as the use of advanced computing sciences (in the fields of big data, genomics and artificial intelligence, for example)”

Although several countries have launched interim guidance on telemedicine, a sustainable telehealth ecosystem would need to take into consideration standards, interoperability, and regulatory frameworks (see [4-6] for further details).

In the subsequent sections, we provide a brief historical overview of telemedicine, with respect to the periods prior to and during the COVID-19 pandemic, in some of the participating countries, particularly India. We then discuss the current status, with respect to both policy frameworks and actual practice in some of the countries represented herein. We further elaborate on the perspectives, as described by different panelists, and suggest the way forward for the adequate adoption and diffusion of telehealth in countries of South Asia and beyond.

**Table 1:** Panelists and their affiliations.

Name and role	Country	Affiliation
Ashvini Goel (Chair)	India	Telemedicine Society of India (TSI), Lucknow
Alexander Thomas (Cochair)	India	Association of Healthcare Providers India (AHPI), New Delhi
Oommen John (Moderator-1)	India	George Institute for Global Health, University of New South Wales, New Delhi, and Prasanna School of Public Health, Manipal Academy of Higher Education, Manipal
A Thanga Prabhu (Moderator-2)	India	St Johns Health Innovation Foundation, Bengaluru
Sunil Shroff (Panelist)	India	Madras Medical Mission, Chennai
Fazilah Allaudin (Panelist)	Malaysia	Planning Division, Ministry of Health
Chaminda Weerbaaddana (Panelist)	Sri Lanka	Ministry of Health
Dari Alhuwail (Panelist)	Kuwait	Information Science Department, Kuwait University, and Health Informatics Unit, Dasman Diabetes Institute
Udaya Koirala (Panelist)	Nepal	Telemedicine Society of Nepal, Kathmandu
JA Jayalal (Panelist)	India	Indian Medical Association, New Delhi
Patricia Codyre (Panelist)	SEARO^a^, World Health Organization	Digital Health and Innovation, SEARO, New Delhi
Andy Bleaden (Panelist)	European Union	European Connected Health Alliance (ECHAlliance), United Kingdom
SN Sarbadhikari (Panelist)	India	George Institute for Global Health, New Delhi
Shubnum Singh (Panelist)	India	Confederation of Indian Industries, National Healthcare Council, New Delhi
Shuchin Bajaj (Panelist)	India	Ujala Cygnus Healthcare Services, New Delhi

^a^SEARO: World Health Organization Regional Office for South-East Asia.

## Brief Historical Overview

### Pre-COVID-19 Period

Telemedicine has been helping family physicians by giving them easy access to specialized physicians and helping them in the close monitoring of patients. Various types of telemedicine services such as store and forward, and real-time, remote, and self-monitoring provide various educational, health care delivery and management, disease screening, and disaster management services across South Asian countries. In India, telemedicine had been traditionally led by some like-minded and passionate health care experts from diverse backgrounds and organizations, who, in their quest to bridge the humongous “health care divide” in India, decided to utilize communication technology for the provision of quality health care to care-seekers in underserved and difficult-to-reach areas of the country. They were ably facilitated by the Indian Space Research Organization with offer of their satellites for the purpose, since 2001. In India, several public and private telemedicine projects have already been in place, although fragmented and at small scales [7].

In Nepal, rural and remote health centers have been connected through a telemedicine network for specialist consultation, although issues such as electricity connection and network connectivity have hindered the widespread adoption and diffusion [8].

Sri Lanka has been promoting teleconsultations in response to emergency situations following the tsunami in 2004 [9].

### During (and After) COVID-19

The Indian government released telemedicine practice guidelines [10] soon after the global lockdown in 2020 in response to the COVID-19 pandemic to promote teleconsultations. Free teleconsultations are being offered through the government-sponsored *e-sanjeevani OPD* telemedicine platform [11]. The government also partnered with many private organizations to promote teleconsultations.

There is compelling evidence [12] to suggest that telehealth may have a significant effect to advance the health care of the future. Nevertheless, the feasibility and application of telehealth in resource-constrained settings and low- and middle-income countries must be established to avail its potential and transform health care for the global population. As telehealth is advancing rapidly, a global consensus is absolutely necessary for definitions, boundaries, protocols, monitoring, and evaluation, as well as to ensure data privacy.

Telemedicine adoption had accelerated because of COVID-19. From March 2020 onward as the number of cases increased, a WhatsApp group was first used to help prevent the spread of disease. A small telemedicine platform was used to reach more patients. State government accommodations though legal cover came much later. Within 6-7 months, 10,000 volunteers came forward to help without salary or recognition. Real-time triaging of COVID-19 cases has been accomplished in 16 states. Teletriaging has been performed to avoid panic. Fake news and the spread of misinformation (infodemic) also need to be addressed [13,14]. During the onset of the pandemic in India, less tests were conducted, and sometimes the reports of COVID-19 tests were not shared with the patients in a timely manner [15]. Social isolation, home quarantine, or institutional isolation were implemented and patients were appropriately advised. Plasma use went into the black market and donors were difficult to find initially, although this was solved over time. No charges were collected from users, and some organizations had been running COVID-19 response teams with grants only.

Experts also stressed the importance in addressing interoperability needs beyond technological aspects. Interoperabilities for human and institutional factors such as culture, governance, and policy also need to be kept in mind. For addressing these issues, changes in management principles have to be applied judiciously and continuously. The National Digital Health Mission and the Swasth alliance have come together to help manage COVID-19. The novel coronavirus (SARS-Cov-2) has globally acted as the chief transformation officer, causing massive digital disruption, especially for the health and education sectors. Learning from each other, we should be able to address the bigger problem. Digital health literacy is also badly needed. Digital determinants of health should be addressed [16]. Catching them at a young age would make health care workers more digitally savvy. The capacity building of human resources for health needs to be implemented in an ethical manner to enhance patient safety [17].

In Nepal, the government actively promoted the use of information and communications technology during the pandemic by offering several online consultation apps, and telemedicine practice guidelines were released [18].

In Sri Lanka, a robust primary health care delivery model along with a strong telecommunication network supported health care delivery during COVID-19 [9].

## Current Status

### Policy

In India, the Telemedicine Practice Guidelines 2020 were formally notified in May 2020 [10]. Although various other countries have been trying to promote telemedicine since the turn of the millennium, digital disruption enforced by the COVID-19 pandemic has hastened and streamlined these efforts. In general, all countries in this region have been supportive and encouraging toward the adoption of telehealth across the continuum of health care delivery.

The oil-rich Gulf Region, including the state of Kuwait, has made huge strides in the adoption of digital health solutions, including telehealth, electronic health records, laboratory information systems, picture archiving and communications systems, radiology information systems, and health information exchange in some countries [19]. However, challenges exist with medical terminologies and adoption of various standards for these digital solutions. With respect to telehealth specifically, it was pointed out that experiences shared by experts from normative agencies such as the World Health Organization (WHO) [20-22] and other groups such as the TSI should be shared with the community for learning purposes. One utterly important fact that was stressed is the safety of digital technology, which is paramount, while keeping patients or consumers of health services in the center of care and empowered to play a key role. The discussion also focused on the importance of establishing legal and ethical frameworks that are respectful of various cultures throughout the care continuum.

### Actual Practice

Experts from Malaysia [23] stressed upon the fact that rather than highlighting new technologies only, people and processes need to come together. Telemedicine is only a medium. Privacy and confidentiality are important. Patients’ and consumers’ digital rights must be respected.

In Sri Lanka, physicians are trained at University of Colombo with an MSc in health informatics, which has given rise to a talent pool that has been very useful to deploy telemedicine. Training of health care workers [24] was essential for success. Chaminda Weerbaaddana from the Ministry of Health, Sri Lanka, stated:

Sri Lankans have access to a primary health care organization within a very short distance irrespective of their geographical location of residence. As such, before the pandemic, provider to client telehealth was not seen as a priority. Despite lack of need for telehealth services due to geographical reasons, there was a demand for such services due to diseases associated with stigma, where people would prefer to maintain anonymity when seeking services.

Legal cover is essential to practice telemedicine. Guidelines have already been put in place by the Ministry of Health. More than 200 physicians have been trained in telemedicine. Two vendors have been identified and services are offered free to the public. Training was conducted online. Thus, Sri Lanka has proven that health care can be delivered via telemedicine, as has been done for COVID-19. Training care providers along with private players, especially with respect to ethics and security issues, is being undertaken.

Although Kuwait [19] is a small nation, it has a huge diversity in its population across socioeconomic status, language, and culture. This diversity needs to be considered when trying to build a standardized telemedicine practice, irrespective of service provider or consumer background. Dari Alhuwail, from Kuwait, stated:

Telemedicine should be integrated with national health strategies and those investments need to be made not only in equipment, but in training the workforce and ensuring continuing support. All digital health solutions need to be humanized and a dialogue amongst all stakeholders to tailor the solutions to serve them all is essential.

It was also suggested that digital health should be part of medical and health sciences educational curricula, and potentially even in the general and higher education systems where consumers of health services can understand how to best leverage these tools and play a more active role in their own health care.

In Nepal, new technology has to be adopted. The human factor may be one of the major barriers for the speedy development and use of telemedicine. Doctor-to-doctor and doctor-to-patient consultations are quite different. Based on a surgeon’s experience, it was stated that cameras that are used to cover remote surgery have been found to be useful. Demystification of telemedicine technology should be obtained through vigorous training for all levels of human resources involved. A structured telemedicine curriculum should be introduced to formal medical and information technology education at different levels. Continued technical support at remote sites is necessary for continued service and to avoid unnecessary frustration.

The WHO South East-Asia Regional Office has shared WHO digital health guidelines and specific guidance on telemedicine implementation that have been developed in consultation with member states [20,21]. WHO guidelines aligned to sustainable development goals (SDGs) and digital health platforms for tracking progress on health-related SDGs are also under implementation to track triple-billion targets. A digital health implementation handbook was recently released during the virtual World Health Assembly. These frameworks provide the building blocks for telemedicine. COVID-19 has served as a gentle push to take health care online. Following these recommendations, judicious roadmaps are a key to success. Patient centricity is essential. Improving eHealth awareness is needed. Immediate response to the current pandemic is essential, but we also need to learn from it quickly. Human capacity needs to be built up, and digital tools and telehealth should be leveraged for capacity building.

In India, industry bodies such as the Federation of Indian Chamber of Commerce and Industries and Confederation of Indian Industries, in collaboration with the Health Sector Skills Council, could contribute to the mainstreaming of telehealth in India.

The Indian Medical Association (IMA) is in favor of the widespread use of telehealth in India. India has 1,062,398 modern medicine practitioners registered with medical councils as of December 31, 2017 [22]. Sensitizing and educating such a large number of doctors in India can be a daunting task. To address the issue, a telehealth training course called “Train to Practice” was designed by the TSI [21]. The TSI initiated this training for registered medical practitioners (RMPs) within 2 weeks of the guidelines having been passed. The volunteer members of the society (including some coauthors of this article) trained close to 3000 doctors and sensitized another 25,000 doctors within the next 6 months [25]. This course is now available online for all RMPs [26].

Even in Europe, the value of health care ecosystems is now being understood in the context of the vulnerabilities that the pandemic has exposed. Health and social care players are coming together and silos are being broken. With a transforming health care delivery system, new economic opportunities are emerging. The European Connected Health Alliance (ECHAlliance) shared how connecting the dots [27,28] can help to scale up innovation built in Australia to be rolled out across Scotland and then to Ireland, which can then be showcased back to Australia where they have shared their best practice in a proven deployment. Andy Bleaden from ECHAlliance stated:

Our ECHAlliance Ecosystems connected the dots in health care during the pandemic taking solutions from one country and adopting them nationwide in another as the NHSNearMe program in Scotland and then offering this adoption back to the Australian health care market who had not seen it implemented at scale.

## Perspectives

Textbox 1 summarizes the current status of telehealth implementation according to the discussant countries, along with the next steps and barriers to implementation.

**Next steps and barriers to telehealth implementation.**

**India**

***Barriers***

- Privacy and security
- Perceived ease of use
- Language barriers
- Fraud and abuse
- Questionable quality of care
- Perceived usefulness/preference for face-to-face consultation
- Shortage of tech-savvy workforce
- Inappropriate behavior by patients
- Digital divide
- Institutional, cultural, and governance issues
- Medical errors

***Next steps***

- The Personal Data Protection (PDP) Bill, currently tabled in the Parliament, may soon be passed to give robust directions for policy and implementation
- Appropriate capacity building for human resources for health and for raising awareness for patients may be undertaken
- With the imminent 5G connectivity in India, the digital divide is likely to be reduced
- Health data literacy and digital health literacy need to be encouraged across the entire health professional education environment
- The National Digital Health Mission is likely to ensure the smoother adoption of digital health

**Sri Lanka**

***Barriers***

- Inadequate training of health care providers
- Privacy and security
- Ethical issues

***Next steps***

- Training the care providers along with private players, especially for ethics and security issues, is being undertaken

**Kuwait**

***Barriers***

- Huge diversity in the population across socioeconomic status, language, and culture
- Inadequate training of health care providers
- Lack of user friendliness of digital health solutions

***Next steps***

- Telemedicine needs to be integrated with national health strategies
- Investments need to be made in training the workforce and ensuring continuing support
- All digital health solutions need to be humanized and a dialogue among all stakeholders to tailor the solutions to serve them all is essential

**Malaysia**

***Barriers***

- People and processes need to come together
- Privacy and confidentiality are important
- Patients’ and consumers’ digital rights must be respected

***Next steps***

- The Malaysian Medical Council Advisory on Virtual Consultation (2020) defined the clinical, ethical, legal, technical, and operational aspects of telemedicine for health practitioners

**Nepal**

***Barriers***

- The human factor may be one of the major barriers for the speedy development and use of telemedicine; doctor-to-doctor and doctor-to-patient consultations are different
- Regular technological support, particularly at remote sites, is often unavailable

***Next steps***

- Demystification of the telemedicine technology must be obtained through vigorous training for all levels of human resources involved
- A structured telemedicine curriculum should be introduced to formal medical and information technology education at different levels
- Continued technical support at remote sites is necessary for continued service and to avoid unnecessary frustration

**European Union**

***Barriers***

- Underestimating the value of health care ecosystems
- Health care and social workers working independently
- Doubts regarding scale and economic outcomes

***Next steps***

- The value of health care ecosystems is now being understood in the context of the vulnerabilities that the pandemic has exposed
- Health and social care players are coming together and silos are being broken
- With a transforming health care delivery system, new economic opportunities are emerging. The European Connected Health Alliance (ECHAlliance) is connecting the dots to help scale up innovation built in Australia to be rolled out across Scotland, followed by Ireland, and then showcased back to Australia

## Conclusions

All panelists emphasized the need for active encouragement toward the adoption and diffusion of digital health in general and of telehealth in particular. All stakeholders, ranging from governmental policymakers to common citizens, have to come together to build trusting partnerships to realize the advantages offered by telehealth. The panelists emphasized the importance of scientific research and evidence-based policy recommendations to improve the use and adoption of effective, efficient, and safe digital health solutions. Various telemedicine applications were referred to, such as those used for diagnosing surgical site infections [29] as well as for rural use in the context of the COVID-19 pandemic [30].

After summarizing the proceedings, the Chair suggested that we can develop appropriate courses for telehealth and introduce them as a part of the curriculum for all health professionals, including physicians, surgeons, dentists, nurses, and allied health care professionals. Like-minded organizations such as the Association of Healthcare Providers of India, TSI, IMA, and reputed academic institutions can come together and influence the regulatory commissions for adopting these recommendations.

## Abbreviations

ECHAlliance: European Connected Health Alliance
GSDH: Global Strategy for Digital Health
IMA: Indian Medical Association
RMP: registered medical practitioner
SDG: sustainable development goal
TSI: Telemedicine Society of India
WHO: World Health Organization
